# Influenza Surveillance and Incidence in a Rural Area in China during the 2009/2010 Influenza Pandemic

**DOI:** 10.1371/journal.pone.0115347

**Published:** 2014-12-26

**Authors:** Ying Zhang, Lin Li, Xiaochun Dong, Mei Kong, Lu Gao, Xiaojing Dong, Wenti Xu

**Affiliations:** 1 Department of Infectious Disease, Tianjin Centers for Disease Control and Prevention, Tianjin, China; 2 Institute of Pathogenic Microbiology, Tianjin Centers for Disease Control and Prevention, Tianjin, China; 3 Hangu Centers for Disease Control and Prevention, Binhai New Area, Tianjin, China; The University of Hong Kong, Hong Kong

## Abstract

**Background:**

Most influenza surveillance is based on data from urban sentinel hospitals; little is known about influenza activity in rural communities. We conducted influenza surveillance in a rural region of China with the aim of detecting influenza activity in the 2009/2010 influenza season.

**Methods:**

The study was conducted from October 2009 to March 2010. Real-time polymerase chain reaction was used to confirm influenza cases. Over-the-counter (OTC) drug sales were daily collected in drugstores and hospitals/clinics. Space-time scan statistics were used to identify clusters of ILI in community. The incidence rate of ILI/influenza was estimated on the basis of the number of ILI/influenza cases detected by the hospitals/clinics.

**Results:**

A total of 434 ILI cases (3.88% of all consultations) were reported; 64.71% of these cases were influenza A (H1N1) pdm09. The estimated incidence rate of ILI and influenza were 5.19/100 and 0.40/100, respectively. The numbers of ILI cases and OTC drug purchases in the previous 7 days were strongly correlated (Spearman rank correlation coefficient [*r*] = 0.620, *P* = 0.001). Four ILI outbreaks were detected by space-time permutation analysis.

**Conclusions:**

This rural community surveillance detected influenza A (H1N1) pdm09 activity and outbreaks in the 2009/2010 influenza season and enabled estimation of the incidence rate of influenza. It also provides a scientific data for public health measures.

## Introduction

In China, urban general hospitals carry out influenza-like illness (ILI) surveillance. Participating institutions include urban pediatric hospitals, specifically pediatric internal medicine outpatient, emergency, and fever clinics. Sentinel hospitals (secondary, tertiary, and pediatric urban hospitals) collect throat swab specimens from patients with ILI weekly to carry out laboratory surveillance. However, this surveillance likely does not capture residents of villages and townships, who typically visit primary hospitals or village clinics when presenting with ILI [1], [2]. Moreover, many patients with ILI purchase medicine at local pharmacies instead of visiting a doctor; 53% of these patients purchase over-the counter (OTC) medications [3], [4]. In this study, we thus conducted influenza surveillance of the whole population in primary hospitals and village clinics in a rural area of China with the aim of detecting influenza activity in the 2009–2010 season. Surveillance included ILI, laboratory-confirmed influenza cases, and OTC drug sales. Outbreak detection was carried out by applying temporal and spatiotemporal statistical methods, and the incidence rate of influenza was also estimated.

## Materials and Methods

### Study site and population

The study sites were two townships and six villages located in rural Tianjin, northeastern China, approximately 30 km from Tianjin City. This area has a typical temperate climate with an influenza season from October to March. We carried out influenza surveillance in the 2009/2010 influenza season in two primary hospitals and six village clinics that provide services only to residents living in these communities. The region has a total of 40,000 inhabitants, with an 1157 (range, 750–2097) residents per community.

### Percentage and incidence rate of ILI

ILI is defined as the presence of fever (≥38°C) and cough and/or sore throat in the absence of any other laboratory-confirmed diagnosis [5]. The percentage of visits for ILI (ILI%) was defined as the number of ILI cases among the total number of consultations:



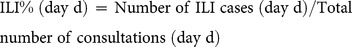


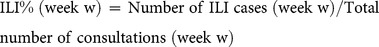



Physicians in primary hospitals and village clinics diagnosed ILI cases and reported them by age group (0–4, 5–14, 15–24, 25–59, ≥60 years). Designated hospital staff reported the number of ILI cases by age group and total number of consultations in these hospitals and clinics daily on line [6].

The incidence rate of ILI/influenza was estimated on the basis of the number of ILI/influenza cases detected by the hospitals/clinics, considering the proportion of the census population who seek care in the sites when got ILI symptom. For this purpose, we designed a questionnaire to investigate the proportion in 430 residents in the community. The proportion of ILI visiting a hospital/clinic in the population was 20.9%.

Incidence rate of ILI/influenza (year y)  =  Number of ILI/influenza cases (year y)/the general population number(2010 census year)/0.209*100.

### Laboratory methods

Attending physicians or nurses collected throat swabs from all patients with ILI. Demographic and syndrome information was also collected. The district-level Centers for Disease Control and Prevention (CDC) obtained specimens from hospitals and clinics daily. Specimens were transported to the influenza laboratory at 4°C within 48 h of collection. Real-time reverse-transcriptase polymerase chain reaction (RT-PCR) assays were used for influenza subtype detection.

### Over-the-counter drug sales

We monitored the purchase of OTC medications at the two primary hospitals and two largest of five drugstores in the region. We collected daily data on the number of visitors buying influenza-related OTC drugs by pharmacies' sales records, including adult and pediatric fever/cold, cough, and nasal medications in liquid or tablet form.

### Outbreak surveillance

Space-time permutation scan statistics (SaTScan software) were used to identify ILI clustering in the eight communities. This statistic utilizes thousands or millions of overlapping cylinders representing potential outbreak candidates to define the scanning window [7]. Each cylinder's height and base represented the number of days and geographic area, respectively. Cylinders were centered on hospital/clinic using latitude and longitude. ILI cases were aggregated by village based on the hospital/clinic address. The Poisson generalized likelihood ratio (GLR) was used within the space-time permutation model to measure the evidence that a given cylinder contained an ILI outbreak. GLR is defined as the observed divided by the expected to the power of the observed inside the cylinder, multiplied by the observed divided by the expected to the power of the observed outside the cylinder [7]. The cylinder with the maximum GLR was considered to represent the space-time cluster of cases that was least likely to be a chance occurrence. Monte Carlo hypothesis testing was used to evaluate the statistical significance of the window. As we performed 999 Monte Carlo replications, the smallest p-value was 0.001. Analyses were adjusted for space by daily interaction [7].

### Ethics statement

The Institutional Review Board of Tianjin CDC approved the design and protocol of this surveillance study, which was conducted in accord with the amended Helsinki Declaration. All data were anonymized upon collection or prior to analysis. Throat-swab specimen collection and case investigation were performed after trained surveillance doctors explained the purpose of the study and obtained verbal and written consent to study participation from patients or their parents or guardians.

## Results

### ILI surveillance

**Figure 1:**
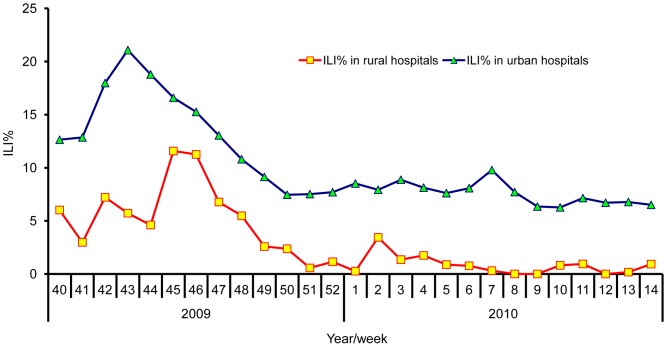
Comparison of weekly ILI% between rural area and urban area, China, 2009/2010 influenza season.

In the influenza season extending from October 2009 to March 2010, surveillance hospitals/clinics reported detection of 434 cases of ILI during 11,198 consultations (ILI% = 3.88%). This regional ILI% was strongly correlated with the ILI% determined by urban-level sentinel surveillance during this period (Spearman rank test, *r* = 0.675, *P* = 0.000; Fig. 1).

### Laboratory findings

**Figure 2:**
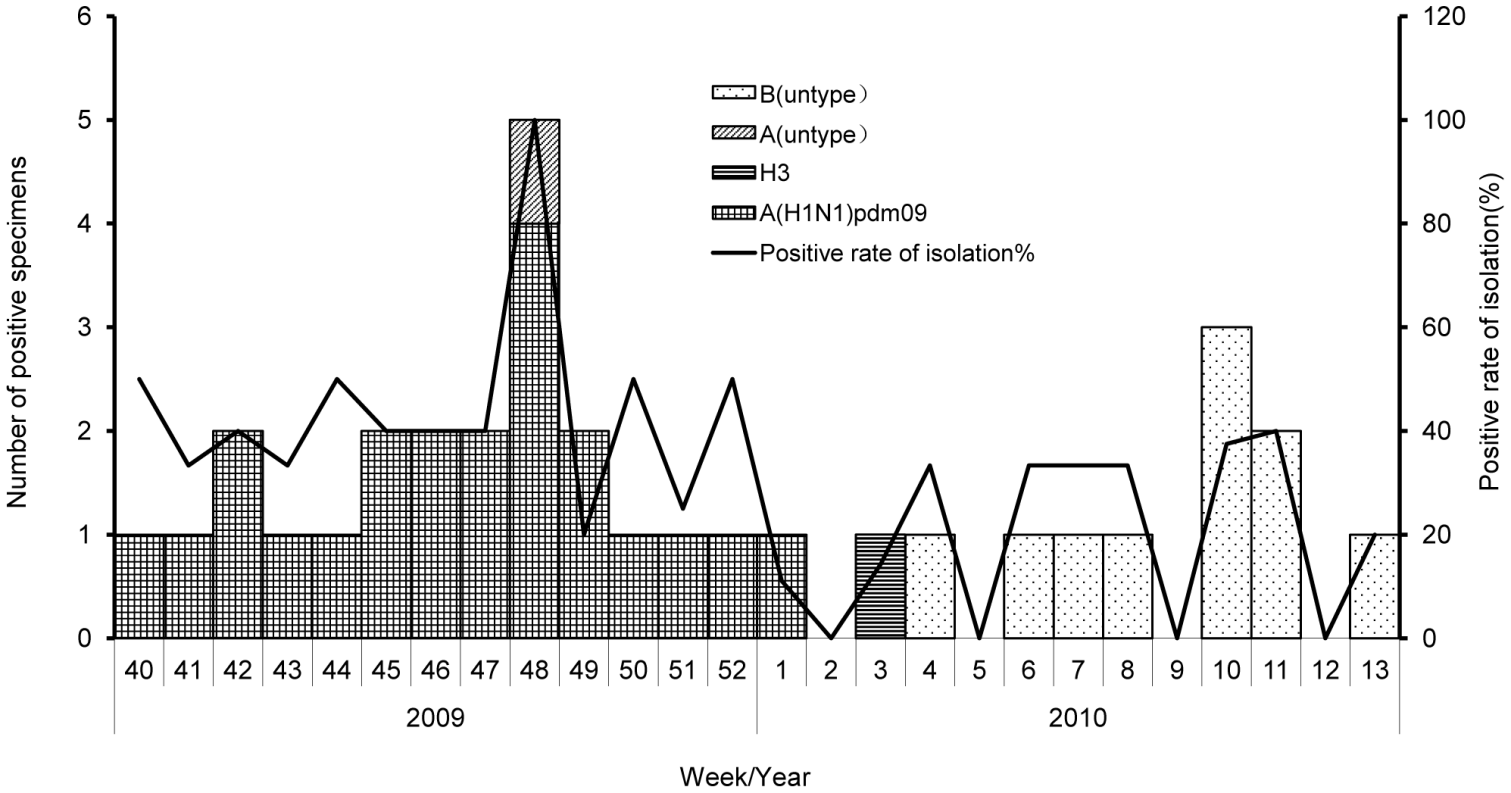
Influenza virus isolations among influenza-like (ILI) cases in a rural area, China, 2009/2010 influenza season.

All 434 ILI cases were subjected to laboratory analysis. Thirty-four (7.83%) cases showed positivity for influenza virus by RT-PCR. Influenza A (H1N1) pdm09 was identified in 22 (64.71%) of these cases, the influenza B subtype was detected in 11 (32.35%) cases, and subtypes H_3_ and A were detected in one case each (Fig. 2).

### Over-the-counter drug purchasing

**Figure 3:**
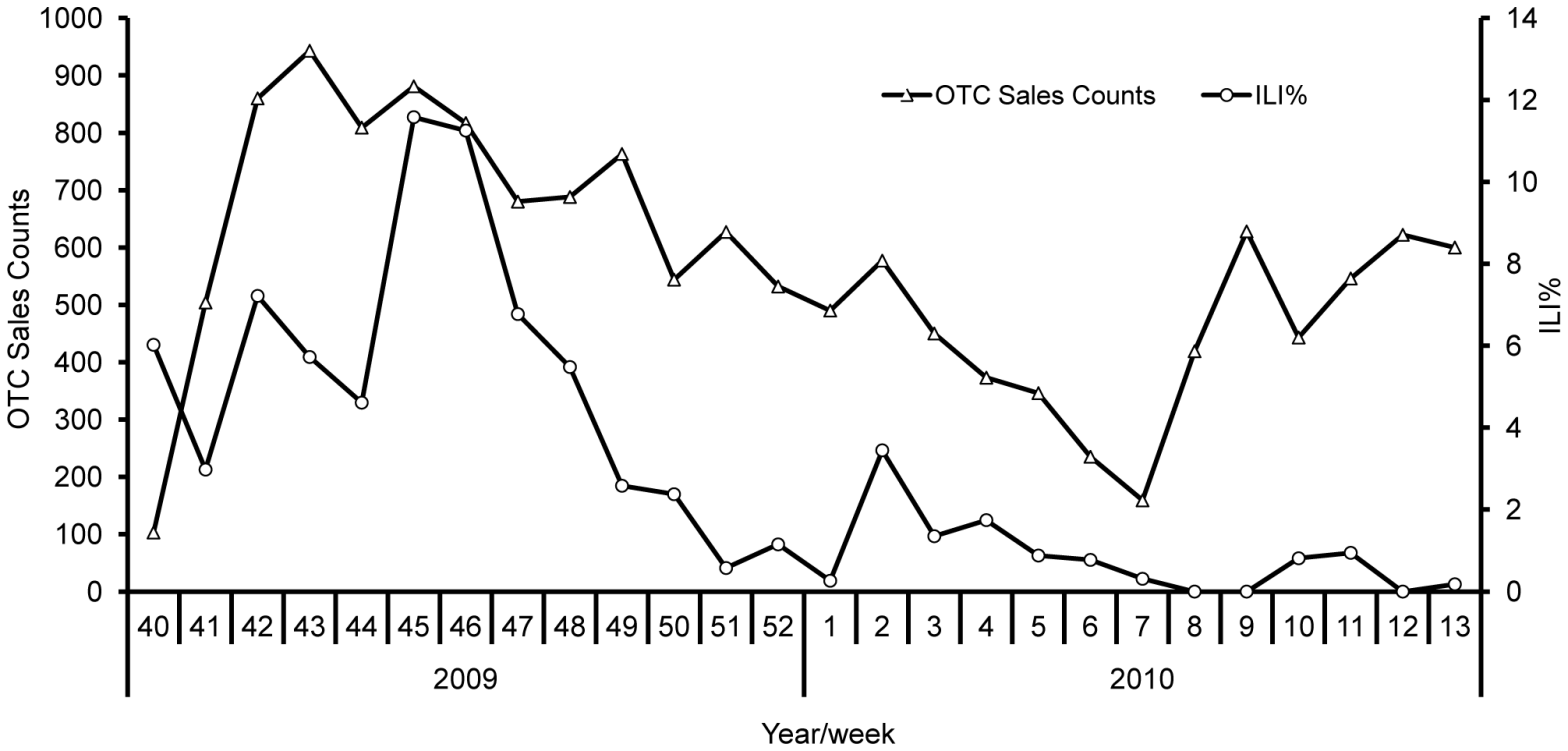
Comparison between ILI% and OTC drug sales in a rural area, China, 2009/2010 influenza season.

From October 2009 to March 2010, 14,333 purchases of at least one OTC medication under investigation were recorded. OTC drug sales peaked at 170 customers per day in October 2009, then fluctuated and decreased until March 2010, when the peak was 120 customers per day (Fig. 3). The numbers of ILI cases were strongly correlated with OTC sales in the previous 7 days during this period (*r* = 0.620, *P* = 0.001; Fig. 3).

### Outbreak surveillance

**Figure 4:**
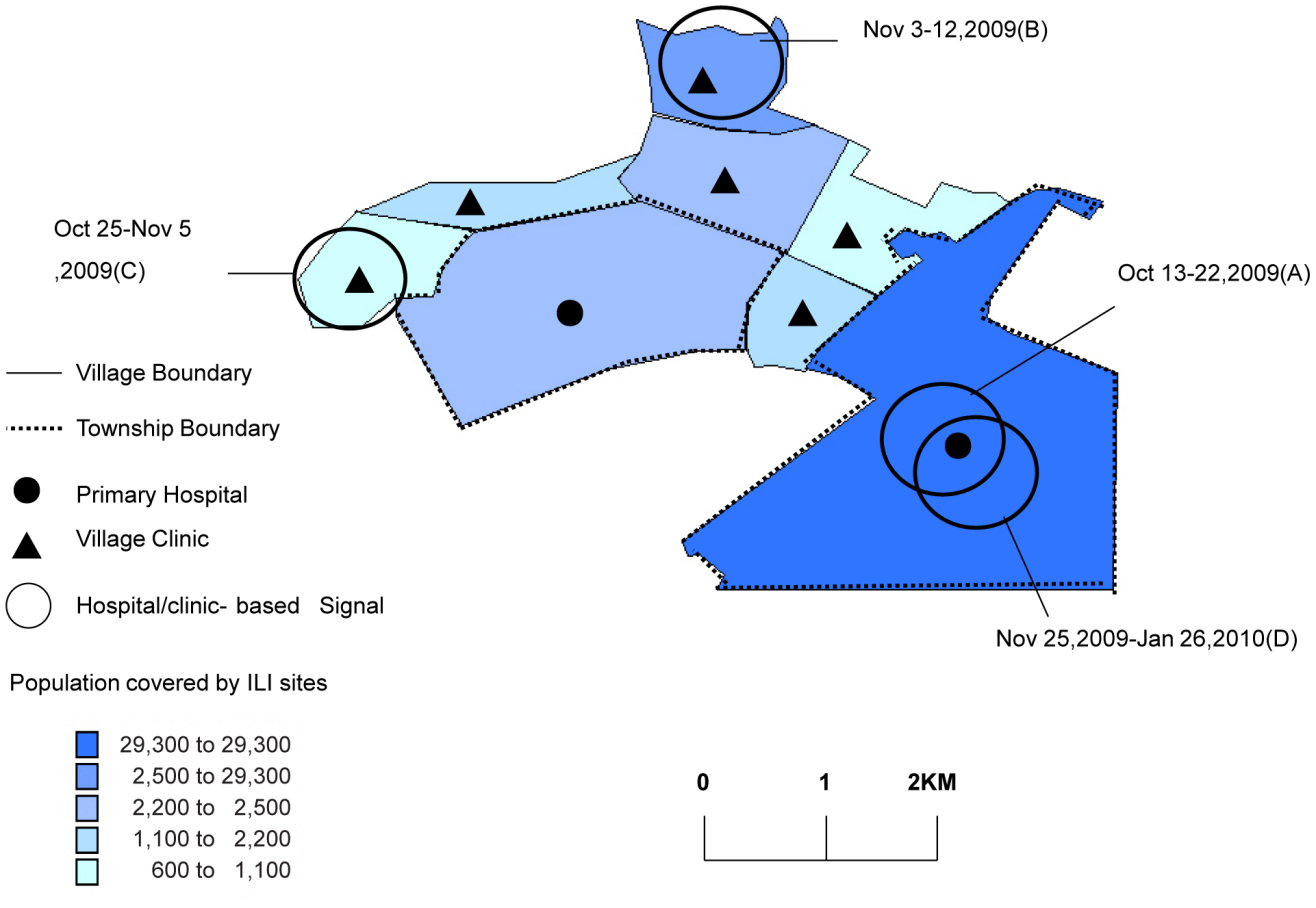
ILI cluster detection by using a space-time permutation model in a rural area, China, 2009/2010 influenza season.

The space-time permutation model detected four significant spatiotemporal clusters of ILI during the 2009/2010 influenza season. Investigation of these locations led to the identification of influenza A (H1N1) pdm09 outbreaks in communities on 12–22 October 2009, 3–12 November 2009, 25 October–5 November 2009, and 25 November 2009–26 January 2010 (Table 1, Fig. 4).

**Table 1:** The space time clusters of ILI in a rural Community, China, 2009/2010 influenza season.

Signals	Date of cluster	observed cases	Expected cases	Relative risk	P value
A	2009.10.13–22	70	26.59	27.44	0.001
B	2009.11.3–11.12	77	42.58	13.23	0.001
C	2009.10.25–11.5	21	6.52	10.4	0.001
D	2009.11.25–2010.1.26	53	28.43	9.99	0.001

### Estimation of ILI/influenza incidence rate

The incidence rate of ILI and influenza, based on a 20.9% visiting rate from October 2009 to March 2010, were 5.19/100 inhabitants and 0.40/100 inhabitants, respectively (Table 2).

**Table 2:** The incidence rate of ILI/influenza in general population from October 2009 to March 2010.

Year	Month	ILI	ILI incidence rate%	Influenza case	Influenza incidence rate%
2009	October	153	1.83	5	0.06
	November	216	2.58	7	0.08
	December	31	0.37	9	0.11
2010	January	21	0.25	3	0.04
	February	4	0.05	3	0.04
	March	9	0.11	6	0.07
Total		434	5.19	33	0.40

Abbreviation: ILI, influenza like illness

The total population number is 40000, the proportion of the census population who seek care in the sites when got ILI symptom was 20.9%.

## Discussion

Sentinel surveillance in China is currently based on a comprehensive general hospital model that does not include the populations of townships and villages. Influenza surveillance in rural populations, as performed in this study, documented epidemic trends of this virus in rural villages and townships [8].It enables the estimation of ILI and influenza incidence rate and description of regional epidemic characteristics, alerts authorities to outbreaks, and provides a scientific data for public health measures. Future studies should examine differences between urban and rural ILI surveillance.

The number of ILI cases documented by our community surveillance peaked in early November 2009. The finding is in agreement with the single peak of the 2009 H1N1 influenza pandemic in November in urban sentinel hospitals (see Fig. 1).

The observed correlation between OTC drug sales and ILI% in this study is consistent with findings from sentinel surveillance (*r* = 0.799, *P* = 0.001) [9]. These data can document regional influenza epidemic trends [10] and are frequently used during the initial phase of illness. Multiple factors can challenge OTC drug sales. Determining the appropriate response to signals is the largest challenge in routine OTC surveillance. No information about medication purchasers is available, and direct investigation with individual pharmacies or consumers is not possible. The detection of community-level clustering according to OTC drug sales is thus difficult [11].

The early detection of disease outbreaks is important to minimize morbidity and mortality through the timely implementation of disease prevention and control measures [7]. Forecasting models are needed to assist policy making and planning of effective health system responses to epidemics. Extensive related research has been conducted using time and/or space detection algorithms (e.g., regression, smoothing, hidden Markov, and wavelet models) to document sudden and gradual outbreaks [12]. We used the prospective space-time permutation scan statistic for regional ILI/influenza outbreak surveillance. We detected four highly unusual clusters of influenza A (H1N1) pdm09. Space-time analysis is used widely in public health practice. For example, previous studies have used it to investigate *Cryptosporidium parvum* infection on dairy farms, influenza and norovirus using telehealth data, meningococcal disease in Niger, and Rocky Mountain spotted fever cases in the United States [13]–[18]. Spatiotemporal analysis of ILI data is a useful complement to regional influenza surveillance. This study provides evidence that influenza surveillance is possible in a community setting. The space-time scan statistic accounts for spatial differences by using a control period from which to establish local baselines for each spatial unit [13]. It is typically used to monitor outbreaks using emergency department data.

The collection of home addresses of patients with ILI during community surveillance enables the detection of influenza outbreaks in villages or regions. However, cluster analysis has some limitations [19]. For instance, workplace-related outbreaks cannot be detected based on home addresses. Workplace surveillance in this community should be conducted in the future [7].

In this study, prospective real-time surveillance included epidemiological analysis, pathogen identification, and OTC drug sales counts based on the entire population of the study area. Surveillance data were collected daily and spatiotemporal analysis enabled the detection of community influenza outbreaks. The incidence rate of influenza was also estimated. These findings provide data for health department to take public health measurements.
